# Interface morphology effect on the spin mixing conductance of Pt/Fe_3_O_4_ bilayers

**DOI:** 10.1038/s41598-018-31915-3

**Published:** 2018-09-17

**Authors:** Thi Kim Hang Pham, Mário Ribeiro, Jun Hong Park, Nyun Jong Lee, Ki Hoon Kang, Eunsang Park, Van Quang Nguyen, Anny Michel, Chong Seung Yoon, Sunglae Cho, Tae Hee Kim

**Affiliations:** 1 Center for Quantum Nanoscience, Institute for Basic Science, Ewha Womans University, Seoul, 03760 Korea; 2 Department of Physics, Ewha Womans University, Seoul, 03760 Korea; 3 Spin Engineering Physics Team, Division of Scientific Instrumentation, Korea Basic Science Institute, Daejeon, 34133 Korea; 4 Division of Materials Science & Engineering, Hanyang University, Seoul, 04763 Korea; 5 KU-KIST Graduate School of Converging Science and Technology, Korea University, Seoul, 02841 Korea; 6 Department of Physics and Energy Harvest Storage Research Center, University of Ulsan, Ulsan, 44610 Korea; 7 Départment de Physique et Mécanique des Matériaux, Institut Pprime, UPR 3346, CNRS-Université de Poitiers-ENSMA, Poitiers, France

## Abstract

Non-magnetic (NM) metals with strong spin-orbit coupling have been recently explored as a probe of interface magnetism on ferromagnetic insulators (FMI) by means of the spin Hall magnetoresistance (SMR) effect. In NM/FMI heterostructures, increasing the spin mixing conductance (SMC) at the interface comes as an important step towards devices with maximized SMR. Here we report on the study of SMR in Pt/Fe_3_O_4_ bilayers at cryogenic temperature, and identify a strong dependence of the determined real part of the complex SMC on the interface roughness. We tune the roughness of the Pt/Fe_3_O_4_ interface by controlling the growth conditions of the Fe_3_O_4_ films, namely by varying the thickness, growth technique, and post-annealing processes. Field-dependent and angular-dependent magnetoresistance measurements sustain the clear observation of SMR. The determined real part of the complex SMC of the Pt/Fe_3_O_4_ bilayers ranges from 4.96 × 10^14^ Ω^−1^ m^−2^ to 7.16 × 10^14^ Ω^−1^ m^−2^ and increases with the roughness of the Fe_3_O_4_ underlayer. We demonstrate experimentally that the interface morphology, acting as an effective interlayer potential, leads to an enhancement of the spin mixing conductance.

## Introduction

In recent years, spin currents have been a topic of intense scientific research because of its potential application in ultra-low power information technologies^1–6^. In this regard, heterostructures of non-magnetic (NM) metals with strong spin-orbit coupling (SOC) and ferromagnetic insulators (FMI) have emerged as a platform where spin currents can be used, among other spin-related applications^7–9^, to probe interface magnetism^10,11^. In NM/FMI bilayers, the coexistence of the spin Hall effect (SHE), inverse spin Hall effect (ISHE), and the magnetic proximity of the FMI leads to the spin Hall magnetoresistance (SMR) effect^12,13^. The SMR effect results from the spin currents generated in the NM metal via SHE that are reflected at the NM/FMI interface and converted back to a charge current via ISHE^14^. This additional contribution to the charge current leads to a characteristic dependence of the resistivity on the magnetization of the FMI. The spin accumulation and the spin mixing conductance (SMC) at the interface govern how efficiently the spins flow across it^12^, and consequently have an important role on the magnitude of the SMR^12,15^. SMR has been demonstrated in Pt/yttrium iron garnet (YIG) bilayers^8,13^, Pt/Co_2_FeSi^16^, Pt/CoFe_2_O_4_^10,11^, Pt/Fe_3_O_4_^15,17^, among others^15,17,18^, with Pt being used as model NM metal due to its large spin Hall angle^19–22^.

Within the SMR framework, maximizing the magnetoresistance requires increasing the efficiency with which spin currents flow across the NM/FMI interface^8,9,12,15,23–27^. In this regard, it has been reported that the SMC of an NM/FMI interface can be enhanced by increasing the magnetic density at the interface. This can be achieved by inserting atomically thin magnetic interlayers^27–29^, changing the elementary composition, or implanting magnetic impurities at the interface^30,31^. It has also been predicted that interface morphology can play a key role in the SMC, with first principle calculations of NM/ferromagnetic (FM) heterostructures indicating a generalized increase of the real part of the SMC, *G*_r_, with increasing roughness^32^. However, there are few reports clarifying the relation between these two parameters^32,33^.

In this work, we present the study of SMR in Pt/Fe_3_O_4_ bilayers at cryogenic temperatures and identify a dependence of the SMC on the interface morphology. To clarify that dependence, we tune the morphology of the Pt/Fe_3_O_4_ interface by controlling the growth conditions of the Fe_3_O_4_ films, including the film thickness, growth technique, and post-annealing processes, while maintaining consistent magnetic and structural properties of the films. The surface morphology of the Fe_3_O_4_ is characterized and quantified using atomic force microscopy (AFM). Field-dependent and angular-dependent magnetoresistance (FDMR and ADMR) measurements support the observation of SMR in the Pt/Fe_3_O_4_ bilayers. Employing the theory of SMR in NM/FMI heterostructures, we determine the real part of the SMC at the Pt/Fe_3_O_4_ interface and demonstrate that the morphology of the Fe_3_O_4_ film plays a determinant role in its magnitude.

## Results

### Fe_3_O_4_ surface morphology and electrical characterization of Pt/Fe_3_O_4_ Hall bars

The fabrication of the Pt/Fe_3_O_4_ Hall bars followed two distinct procedures (see Methods for additional details). In samples A, A1, B, and B1, the Fe_3_O_4_ films were deposited on MgO(100) substrates using oxide-molecular beam epitaxy (oxide-MBE). In sample C, the Fe_3_O_4_ film was deposited using RF-magnetron sputtering on a MgO buffer layer prepared by ultra-high vacuum (UHV)-MBE. The Fe_3_O_4_ films are 4-nm thick for samples A and A1, 6-nm thick for samples B and B1, and 40-nm thick for sample C. Samples A1 and B1 were additionally subject to a post-annealing process at 300 °C for 3 hours to drive inter-grain diffusion and coalescence, resulting in the change of the Fe_3_O_4_ roughness. Sample C was annealed at 400 °C for 2 hours after the deposition of the Fe_3_O_4_. The Pt layers were deposited onto the Fe_3_O_4_ films by UHV-MBE using shadow masking techniques to pattern six-terminal Hall bars with a 50-*μ*m-wide and 400-*μ*m-long channel. The thickness of the Pt films was kept within the spin diffusion length of Pt and minimized down to the experimental limit where the films formed continuous and conductive layers (2 to 3 nm thick).

**Figure 1:**
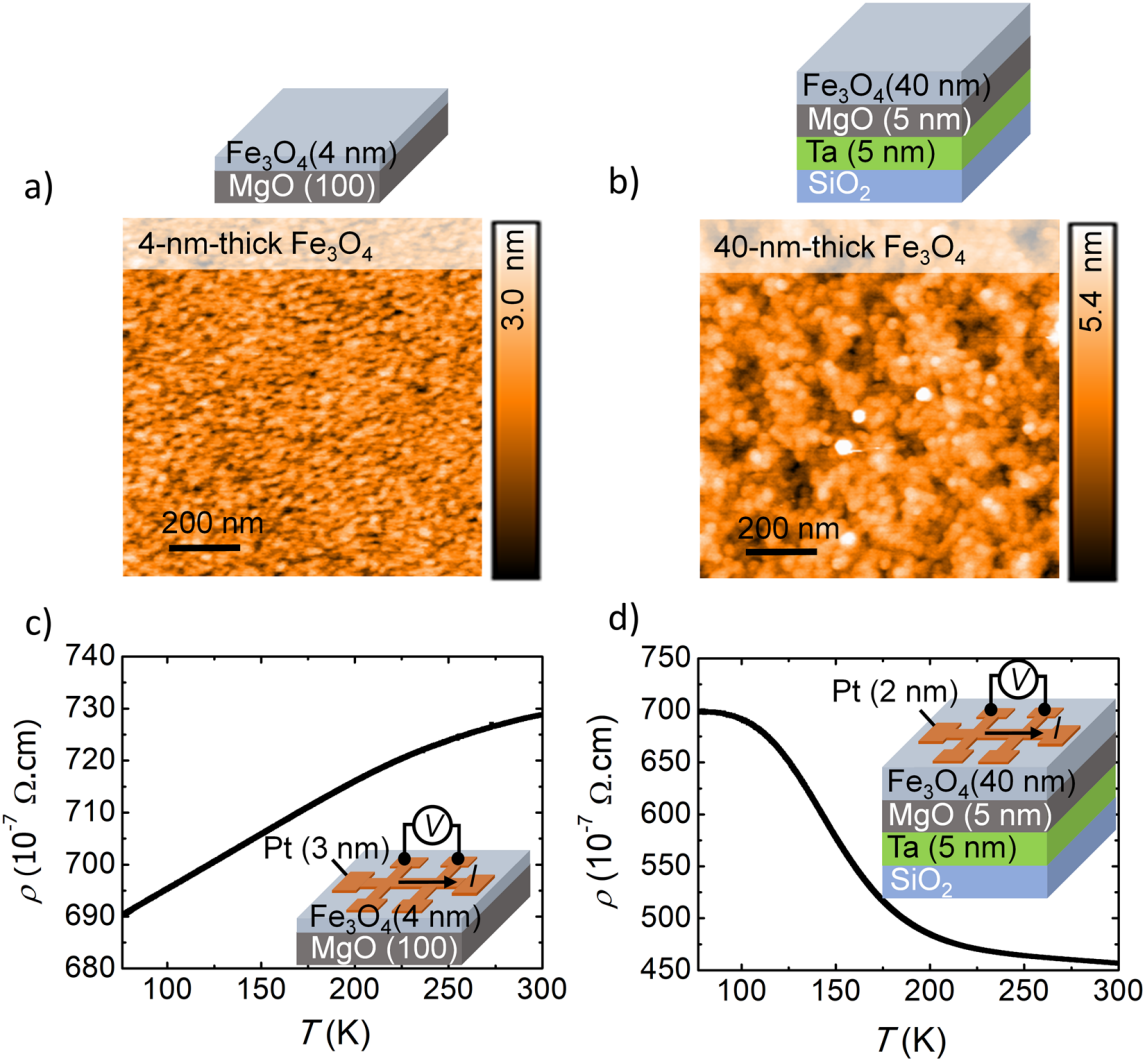
(**a**) AFM 1 × 1 *μ*m^2^ scan of the Fe_3_O_4_ film of sample A and (**b**) sample C at room temperature and environment conditions. (**c**) Temperature dependence of the resistivity of the Pt/Fe_3_O_4_ Hall bar of sample A and (**d**) sample C.

To study the relationship between the interface morphology of the Pt/Fe_3_O_4_ bilayers and the SMR, we started by characterizing the roughness of the Fe_3_O_4_ films using AFM and the temperature dependence of the resistivity of the Pt/Fe_3_O_4_ Hall bars. In Fig. 1a,b, we show AFM scans of the smoothest and roughest Fe_3_O_4_ films, namely of samples A and C, respectively (see Sup. Fig. S1 for the AFM scans of all samples). The AFM scan of the Fe_3_O_4_ film of sample A shows a smooth surface morphology, with root-mean square (RMS) roughness of 0.42 nm, and an average grain size of 32 nm. The AFM image of the Fe_3_O_4_ film of sample C shows a RMS roughness of 1.1 nm and an average grain size of 40 nm. Table 1 summarizes the results obtained using AFM for the five samples. The five different growth conditions (samples A, A1, B, B1, and C) allowed us to achieve a surface roughness of the Fe_3_O_4_ films that ranges from 0.42 nm to 1.1 nm, while maintaining consistent ferromagnetic and structural properties of the films. SMR is sensitive to the structural details at the interface, and ensuring that the films have comparable properties is important for the evaluation of the SMR signals of the different samples. The Fe_3_O_4_ films deposited using both deposition techniques are highly textured and have similar magnetic moment per unit volume (see Sup. Fig. S2 for the magnetic hysteresis loops, and Sup. Fig. S3 for the structural characterization of the films).

**Table 1:** Summary of the growth conditions and surface morphology of the Fe_3_O_4_ films obtained by AFM.

Sample	Structure	Post Anneal.	RMS (nm)	Peak -to- Valley height (nm)	Grain diameter (nm)	Method
A	Fe_3_O_4_[4]/MgO(100)	X	0.42 ± 0.01	3.5 ± 0.1	32.0 ± 1.0	Oxide-MBE
A1	Fe_3_O_4_[4]/MgO(100)	300 °C, 3 hours	0.70 ± 0.03	6.4 ± 0.1	39.0 ± 2.0	Oxide-MBE
B	Fe_3_O_4_[6]/MgO(100)	X	0.52 ± 0.02	5.7 ± 0.7	37.0 ± 1.0	Oxide-MBE
B1	Fe_3_O_4_[6]/MgO(100)	300 °C, 3 hours	0.73 ± 0.02	6.8 ± 0.2	40.0 ± 1.0	Oxide-MBE
C	Fe_3_O_4_[40]/MgO[5]/Ta[5]/SiO_2_/Si(100)	400 °C, 2 hours	1.10 ± 0.01	12.0 ± 1.3	42.0 ± 3.0	RF-magnetron sputtering

The numbers in brackets represent the thickness of the respective layers in nm.

Regarding the electrical characterization of the samples, Fig. 1c,d show the temperature dependence of the longitudinal resistivity, *ρ*, of the Pt/Fe_3_O_4_ Hall bars for the samples with the smoothest and roughest Fe_3_O_4_ films, namely samples A and C. The resistivity measurements were performed from *T* = 300 K down to *T* = 77 K, within the experimentally accessible conditions using liquid nitrogen cooling (see Methods). In Fig. 1c, the 3-nm-thick Pt film of sample A dominates the transport behavior over the 4-nm-thick Fe_3_O_4_ film, with the resistivity of the sample exhibiting a metallic temperature dependence. Sample C (Fig. 1d) exhibits a semiconductor-like temperature dependence because of the extra conduction channels introduced by the 40-nm-thick Fe_3_O_4_ film that compete with the shaped Pt layer (see Sup. Fig. S4 for the temperature dependence of Fe_3_O_4_ films). However, at temperatures close to the Verwey transition of the Fe_3_O_4_ (*T*_V_ = 125 K)^34,35^, the contribution of the Fe_3_O_4_ channel greatly decreases, and Pt dominates the conductance of the sample. For this reason, the resistivity for both cases (samples A and C) converges at ~ 7 × 10^−5^ Ω cm at temperatures below 100 K, on par with the resistivity reported for high-quality Pt films used in SMR experiments of other works^10,15^. The electrical characterization strongly suggests that for the surface roughness present in our samples, 2- to 3-nm-thick Pt layers are enough to ensure the coverage of the surface, forming high-quality, conducting, and metallic films. This is an important feature, since in the framework of SMR the non-magnetic metal thickness should be thin enough to allow for the generation of a spin accumulation at the interface with the FMI. The insulating state of the Fe_3_O_4_ film at low temperatures leads us to focus the study of the SMR at temperatures below the Verwey transition of Fe_3_O_4_, namely at *T* = 77 K.

### Spin Hall magnetoresistance of Pt/Fe_3_O_4_ bilayers

**Figure 2:**
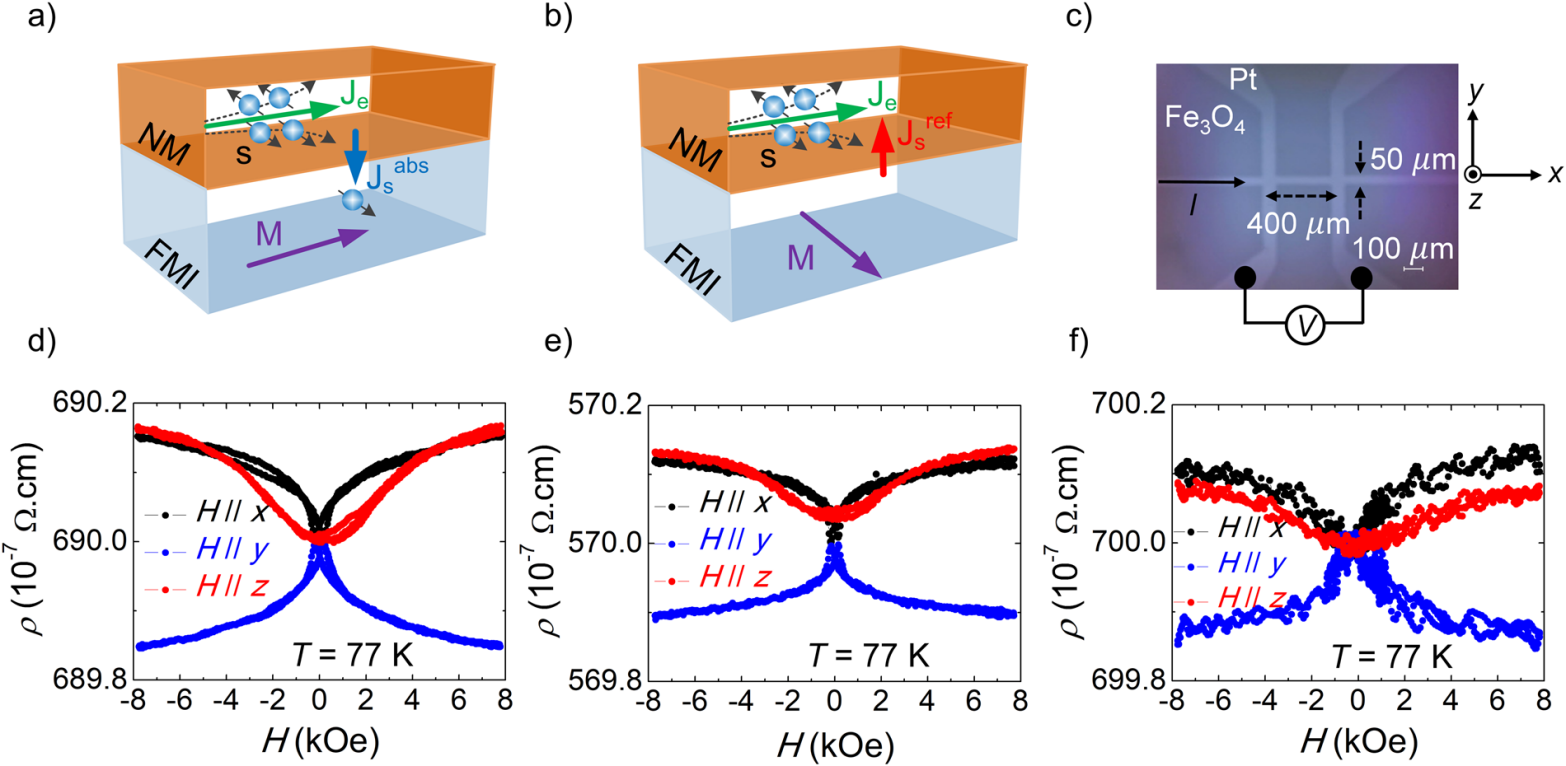
Sketch of the SMR mechanism in a NM/FMI heterostructure. A charge current *J*_*e*_ flowing in the NM with strong SOC is converted into a transverse spin current via SHE, leading to a spin accumulation at the NM/FMI interface. The spin current will be (**a**) absorbed or (**b**) reflected depending on the relative orientation of the magnetization of the FMI and the spin polarization of the spin current. (**c**) Optical microscopy image of the six-terminal Pt Hall bar on the Fe_3_O_4_ layer, with an indication of the *x*, *y,* and *z-*axes. (**d**) Magnetic field dependence of the longitudinal resistivity of sample A, (**e**) sample A1, and (**f**) sample C at *T* = 77 K, for an external magnetic field applied along the *x*, *y,* and *z-*axes.

According to SMR theory, the longitudinal resistivity *ρ* in a NM/FMI bilayer can be formulated as^10,12^

1 $$\rho ={\rho }_{0}+{\rm{\Delta }}\rho (1-{m}_{y}^{2})\,$$

where *ρ*_0_ is a constant resistivity offset, Δ*ρ* is the magnitude of the resistivity change, and *m*_*y*_ is the *y*-component of the unitary magnetization vector, $\hat{{\boldsymbol{m}}}=({m}_{x},{m}_{y},{m}_{z})$, in the direction transverse to the charge current and parallel to the film plane. In Fig. 2a,b, we depict qualitatively the SMR mechanism in a NM/FMI bilayer for the cases when *m*_*y*_ = 0 and *m*_*y*_ = 1, respectively. For *m*_*y*_ = 0, the spin current is transmitted across the interface, and when *m*_*y*_ = 1, the reflected spin current generates an additional charge current via ISHE that decreases the total resistivity^12,14^. In Fig. 2c, we show an optical microscope image of the Pt Hall bar used to determine the dependence of the longitudinal resistivity on the magnetization of the Fe_3_O_4_ underlayer. The current, *I*, of 100 *μ*A, is injected along the Hall bar and the voltage drop, *V*_xx_, is measured at two of the lateral arms along the current path, separated by a length, *L*, of 400 *μ*m. The resistivity is determined from the expression $\,\rho ={V}_{xx}{t}_{NM}W/(LI)$, where *W* is the width of the channel (50 *μ*m). In our samples, we define the *x*-axis parallel to the current injection path, the *z*-axis normal to the film surface, and the *y*-axis perpendicular to both following a right-hand rule.

To confirm the presence of SMR in our samples, we performed FDMR and ADMR measurements. Figure 2d,e, and f show the FDMR of samples A, A1, and C, respectively, at *T* = 77 K, with the external magnetic field applied parallel to the *x*, *y,* and *z*-axes (see Sup. Fig. S5 for the FDMR of samples B and B1). As stated before, at temperatures lower than *T*_V_, the resistivity of Fe_3_O_4_ is higher than the resistivity of Pt^34^, minimizing the current shunting through the Fe_3_O_4_ layer and respective anisotropic magnetoresistance effects^17^. Starting with sample A (Fig. 2d), when the magnetic field is applied parallel to the *x*- and *z*-axis, *H* // *x* and *H* // *z*, respectively, the resistivity increases with the increase of the external magnetic field, saturating in both cases at *ρ* ∼ 690.15 × 10^−7^ Ω cm. Noticeably, when the magnetic field is applied along the *z*-axis, *H* // *z*, the resistivity saturates at higher fields than for the case when *H // x*. This is because of the additional contribution of the shape anisotropy of the Fe_3_O_4_ film. The SMR theory fits well both cases. The decrease of the *y*-component of the magnetization (with the increase of the external magnetic field) is accompanied by an increase of the resistivity, which saturates once the magnetization saturates along the *x* and *z*-axes, as expected from Eq. 1. When the magnetic field is applied along the *y*-axis, *H* //* y*, the resistivity decreases with the increase of the magnetic field, saturating at *ρ* ∼ 689.85 × 10^−7^ Ω cm. Again, this goes in line with the expectation from Eq. 1 that the resistivity should decrease with the increase of the *y*-component of the magnetization, saturating once the magnetization saturates^36,37^. The data obtained from the three FDMR measurements strongly suggests the presence of SMR. Evaluating the data according to Eq. 1, we find for sample A that *ρ*_0_ ∼ 689.85 × 10^−7^ Ω cm and *Δρ*/*ρ*_0_ = 4.6 ± 0.2 × 10^−4^. Similar values have been determined on previous works on Pt/YIG and Pt/Fe_3_O_4_ bilayers^15^. For samples A1 and C, the FDMR is similar to what was observed for sample A, with the remarking difference being the values of *ρ*_0_ and *Δρ*/*ρ*_0_ extracted for both cases. For sample A1, *ρ*_0_ ∼ 569.89 × 10^−7^ Ω cm and *Δρ*/*ρ*_0_ = 4.3 ± 0.1 × 10^−4^, and for sample C, *ρ*_0_ ∼ 699.85 × 10^−7^ Ω cm and *Δρ*/*ρ*_0_ = 4.1 ± 0.2 × 10^−4^. We shall analyze in the Discussion section the implications of these results.

**Figure 3:**
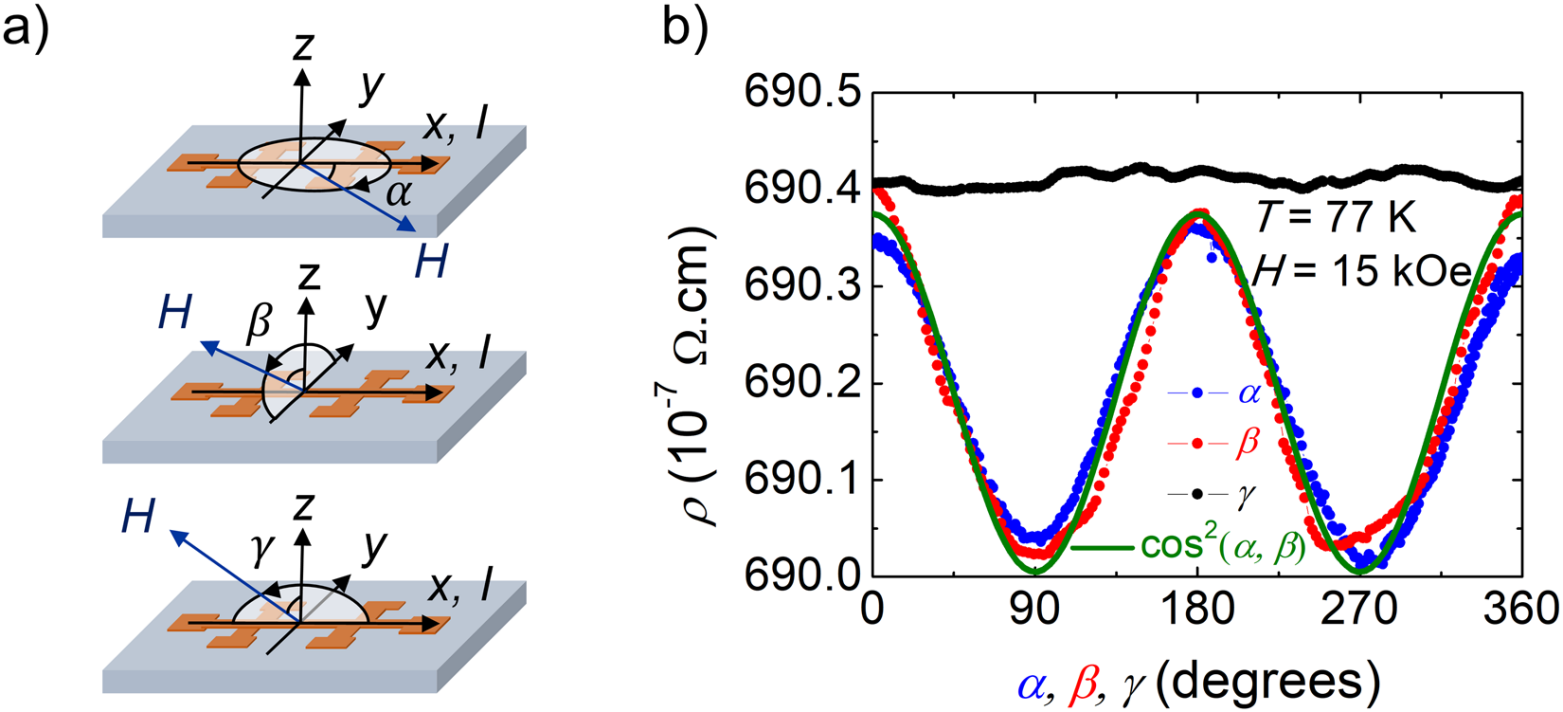
(**a**) Sketch of the three geometries used for the angular-dependent magnetoresistance measurements in the planes defined by the *x-y*, *y-z,* and *x-z* axes, corresponding to angles *α*, *β*, and *γ*, respectively (top-to-bottom). When *α* = 0, the field is aligned with the *x*-axis; when *β*, *γ* = 0, the field is aligned with the *z*-axis. (**b**) Angular dependence of the resistivity of sample A at *T* = 77 K, for a fixed external magnetic field *H* = 15 kOe for the three geometries considered. The green solid line is a guide to the eye for a squared cosine angular dependence.

An additional method to demonstrate and confirm the presence of SMR is to perform ADMR measurements^15,38^. In ADMR measurements, one sets the magnitude of the applied external magnetic field such that the magnetization of the FMI layer saturates in its direction (see Sup. Fig. S2 for the hysteresis loops), and then measures the resistivity as a function of the angle formed between the external magnetic field and the sample. Figure 3a depicts the three different geometries explored, with the rotation of the external magnetic field in the planes formed by the *x*-*y*, *y*-*z*, and *x*-*z* axes quantified by the *α*, *β*, and *γ* angles, respectively (when *α* = 0, the magnetization lies along the *x*-axis, and when *β*, *γ* = 0 the magnetization lies along the *z*-axis). Since the *y*-component of the magnetization should vary only in the cases of an angular dependence in the *x*-*y* and *y*-*z* planes, the angular dependence in the *x*-*z* plane should show no significant change in resistivity. Figure 3b shows the consistency of the experimental data with this argument based on SMR for sample A. The resistivity shows a minimum when the magnetization lies along the *y*-axis (*α*, *β* = 90°, 270°), and a maximum when the *y*-component of the magnetization vanishes (*α*, *β* = 0°, 180°, 360°), with overall dependence proportional to the squared cosine of the angles *α* and *β*, as expected from Eq. 1. Both FDMR and ADMR measurements strongly support the observation of SMR in our Pt/Fe_3_O_4_ bilayers.

## Discussion

**Figure 4:**
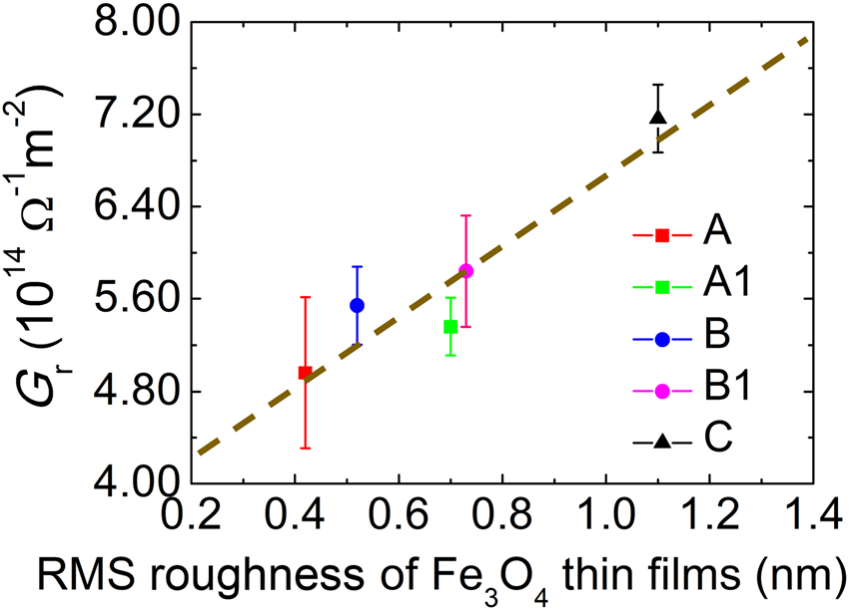
Dependence of the real part of the complex spin-mixing conductance, *G*_r_, on the RMS roughness of the Fe_3_O_4_ thin films. The dashed line is the best linear fit for the data points. Error bars are included.

The SMR ratio *Δρ*/*ρ*_0_ relates to the spin transporting parameters of the NM/FMI bilayer by the expression^12,15^

2 $$\frac{{\rm{\Delta }}\rho }{{\rho }_{0}}\approx {\theta }_{SH,NM}^{2}\frac{\frac{2{\lambda }_{NM}^{2}}{{\sigma }_{NM}{t}_{NM}}{G}_{r}{\tanh }^{2}\frac{{t}_{NM}}{2{\lambda }_{NM}}}{1+\frac{2{\lambda }_{NM}}{{\sigma }_{NM}}{G}_{r}\,\coth \,\frac{{t}_{NM}}{{\lambda }_{NM}}}$$

where *θ*_SH, NM_ is the spin Hall angle, *λ*_NM_ is the spin diffusion length, *σ*_NM_ is the conductivity, *t*_NM_ is the thickness of the NM layer, and *G*_r_ is the real part of the interface SMC. To determine the interface spin transport parameters between Pt and Fe_3_O_4_, one needs a reliable estimate of *λ*_NM_ and *θ*_SH, NM_ of Pt. For this, we followed the work of Sagasta *et al*.^39^ where it is established that the spin relaxation in Pt follows the Elliot-Yafet mechanism, translating into a linear dependence of the spin diffusion length on the conductivity. In the work, the agreement of the spin transporting properties of Pt with the resistivity of devices fabricated with different growth techniques and by different groups provides us the confidence to estimate *λ*_Pt_ and *θ*_SH, Pt_ for our samples. According to the conductivity of our Pt films (between 1.43 × 10^4^ Ω^−1^ cm^−1^ and 2.17 × 10^4^ Ω^−1^ cm^−1^), we estimate the *λ*_Pt_ to range from 1.4 nm to 2.7 nm and *θ*_SH, Pt_ to be ~ 0.056, also in agreement with what was determined by other groups using spin pumping and inverse spin Hall experiments^9^. Using Eq. 1 and Eq. 2, we summarize in Table 2 the SMR data obtained for all the samples considered in this work. The determined *G*_r_ of all samples ranges from 4.96 × 10^14^ Ω^−1^ m^−2^ to 7.16 × 10^14^ Ω^−1^ m^−2^, similar to what has been reported in other NM/FMI bilayers systems, namely Pt/YIG^8,10,13,15,40^ (1.2 × 10^13^ Ω^−1^ m^−2^ to 1.3 × 10^15^ Ω^−1^ m^−2^), Ag/YIG^41^, (4.5 × 10^14^ Ω^−1^ m^−2^), or Au/YIG^42^ (1.9 × 10^14^ Ω^−1^ m^−2^). The dependence of *G*_r_ on the interface roughness becomes clear in Fig. 4, where we plot the calculated *G*_r_ of all samples versus the respective RMS of the Fe_3_O_4_ films determined by AFM. Within the explored RMS, *G*_r_ increases with the increase of roughness. Our data suggests that the reflection/transmission of spins at the interface is significantly altered by the morphology of the Fe_3_O_4_ underlayer. While it has been shown before that the crystalline texture of single crystal FMIs has an effect on the SMC^10,41^, (demonstrating how sensitive the SMC is to the atomic arrangement at the surface), it remained to be demonstrated a clear effect of the surface roughness. Using polycrystalline Fe_3_O_4_ films with grain size orders of magnitude lower than the width and length of the channel of the Hall bars used in this work, we expect the differences in the atomic arrangement at the surface of each individual grain to be averaged out for the whole device. As stated before, interface roughness has been proposed to enhance the SMC in NM/FM heterostructures by introducing an effective potential from a composite NM_1−x_FM_x_ interlayer at the interface^32^. Although we cannot decouple a possible interface-induced spin Hall angle contribution to the SMR^33^, the generalized observation remains that rougher interfaces lead to an increase of *G*_r_.

**Table 2:** Summary of the SMR at 77 K for all samples. *ρ*_0_ and Δ*ρ*/*ρ*_0_ are determined from Eq. 1, and *G*_r_ from Eq. 2. The spin diffusion length, *λ*_Pt_, was estimated based on the resistivity of the Pt films.

Sample	*t*_Pt_ (nm)	*ρ*_0_ (10^−7^ Ω cm)	Δ*ρ*/*ρ*_0_ (10^−4^)	*λ*_Pt_ (nm)	*G*_r_ (Ω^−1^ m^−2^)
A	3.0	689.85	4.6 ± 0.2	1.80	(4.96 ± 0.65) × 10^14^
A1	3.0	569.89	4.3 ± 0.1	2.18	(5.36 ± 0.25) × 10^14^
B	3.0	459.90	3.7 ± 0.3	2.70	(5.54 ± 0.34) × 10^14^
B1	3.0	499.93	4.0 ± 0.4	2.48	(5.84 ± 0.48) × 10^14^
C	2.0	699.85	4.1 ± 0.2	1.77	(7.16 ± 0.30) × 10^14^

In summary, we have demonstrated the SMR effect in Pt/Fe_3_O_4_ bilayers at cryogenic temperatures. From the analysis of the observed SMR, we have shown that the determined *G*_r_ strongly depends on the RMS roughness of the Fe_3_O_4_ film, indicating that the interface morphology of NM/FMI heterostructures is an important parameter contributing to the SMC. Our observations are in-line with previous predictions that the SMC can be enhanced by treating interface roughness as an effective interlayer potential. Since SMC is a critical parameter for various spin-related phenomena in NM/FMI heterostructures, our work suggests that engineering the surface roughness of polycrystalline FMI is a relevant and significant method not only for SMR-based devices, but for device structures that rely on magnetic insulators.

## Methods

### Sample Preparation

Two growth techniques were employed for the deposition of Fe_3_O_4_ films. In samples A, A1, B, B1, the Fe_3_O_4_ thin films were deposited on MgO(100) substrates using oxide-molecular beam epitaxy (oxide-MBE) (VG Semicon, Inc.). The MgO(100) substrates were prepared by cleaning in a bath of methanol at 60 °C and drying with N_2_ gas. Afterward, the cleaned substrates were transferred into the ultra-high vacuum (UHV) chamber, and pre-heated at 600 °C for 30 minutes to remove residual impurities. The substrate was then cooled and maintained at 400 °C for the growth of the thin film. Iron high-temperature effusion cells and oxygen cracking cells were used to grow the Fe_3_O_4_ thin films at a partial oxygen pressure of 10^−6^ Torr. To control the thickness of the films, the evaporation rates were determined using a quartz crystal thickness monitor. The crystal quality during growth was monitored by RHEED (reflection high-energy electron diffraction).

For sample C, the multi-layered architecture was prepared *in-situ* in a home-built UHV-clusters deposition system equipped with MBE and radio-frequency (RF)-magnetron sputtering techniques. The Fe_3_O_4_ thin film was deposited using magnetron sputtering on an MgO buffer layer prepared by UHV-MBE. First, a 5-nm-thick layer of Ta was deposited on a SiO_2_/Si(100) substrate by RF-magnetron sputtering, followed by the growth of a 5-nm-thick layer of MgO using UHV-MBE at a temperature of 200 °C, with a deposition rate of 0.0028 nm/s and at a pressure of 2.5 × 10^−9^ Torr. Afterwards, the sputtering of Fe_3_O_4_ was prepared at a base pressure of 2.3 × 10^−9^ Torr, with a flow of 33 sccm of Ar-gas ensuring a stable plasma. The as-grown Fe_3_O_4_ films were annealed at 400 °C for 2 hours in vacuum.

In all cases, the Pt Hall bars were deposited in an UHV-MBE chamber by electron-beam evaporation at room temperature, using shadow masking, with a deposition rate of 0.016 nm/s, and at a base pressure of 2.0 × 10^−10^ Torr.

### Surface, structural, magnetic, and electrical characterization

The surface morphology, and the magnetic and structural properties of the Fe_3_O_4_ films were characterized using atomic force microscopy (EasyScan2, Nanosurf), vibration sample magnetometry (Quantum Design magnetic property measurement system, MPMS-5XL), and X-ray diffraction and reflectivity (Bruker Discover D8), respectively.

The electrical characterization was performed in a variable-temperature probe station using nitrogen cooling down to 77 K, and with magnetic fields up to 10 kOe. A Keithley 6220 was used as a current source and a Keithely 2182 A as a nanovoltmeter. The angular-dependent magnetoresistance measurements were performed in a Quantum Design physical property measurement system with an in-built low-frequency alternate-current lock-in board. For both cases, standard four-probe measurement schemes were used.

## Electronic supplementary material

Supplementary Information
