# Supplementary material for: Interface morphology effect on the spin mixing conductance of Pt/Fe3O4 bilayers
Source: Sci Rep. 2018 Sep 17;8:13907. doi: 10.1038/s41598-018-31915-3 (PMC6141513; doi:10.1038/s41598-018-31915-3)
Supplement: Supplementary file 1 — Supplementary Information [file 41598_2018_31915_MOESM1_ESM.pdf]

Interface morphology effect on the spin mixing conductance of Pt/Fe_3O_4 bilayers

Thi Kim Hang Pham[1,2], Mário Ribeiro[1,2], Jun Hong Park[1,2], Nyun Jong Lee[3], Ki Hoon Kang[4], Eunsang Park[5], Quang Van Nguyen[6], Anny Michel[7], Chong Seung Yoon[4], Sunglae Cho[6] and Tae Hee Kim[1,2*]

[1]*Center for Quantum Nanoscience, Institute for Basic Science, Ewha Womans University, Seoul, 03760, Korea*

[2]*Department of Physics, Ewha Womans University, Seoul, 03760, Korea*

[3]*Spin Engineering Physics Team, Division of Scientific Instrumentation, Korea Basic Science Institute, Daejeon 34133, Korea*

[4]*Division of Materials Science & Engineering, Hanyang University, Seoul, 04763, Korea*

[5]*KU-KIST Graduate School of Converging Science and Technology, Korea University, Seoul, 02841, Korea*

[6]*Department of Physics and Energy Harvest Storage Research Center, University of Ulsan, Ulsan 680-749, Korea*

[7]*Départment de Physique et Mécanique des Matériaux, CNRS-Université de Poitiers-ENSMA, France*

** Corresponding author. E-mail: taehee@ewha.ac.kr*

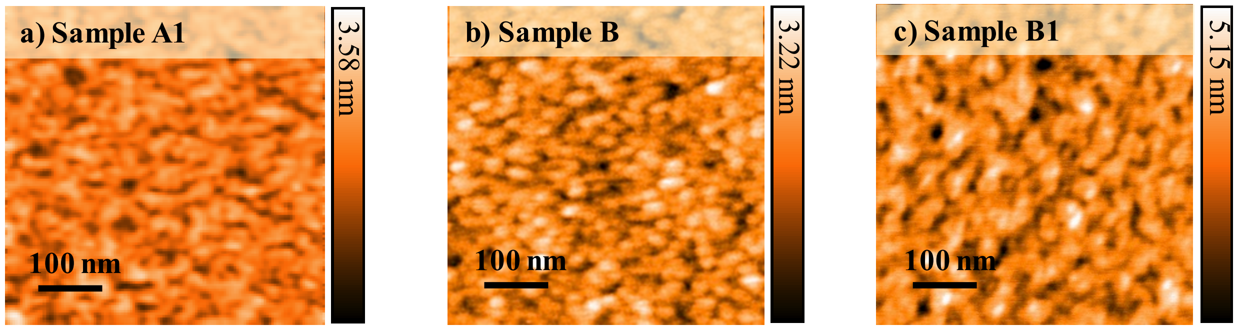

Supplementary Figure S1. (a) AFM 0.5 × 0.5 μm^2 scan of the Fe_3O_4 film of sample A1, (b) sample B, and (c) sample B1.

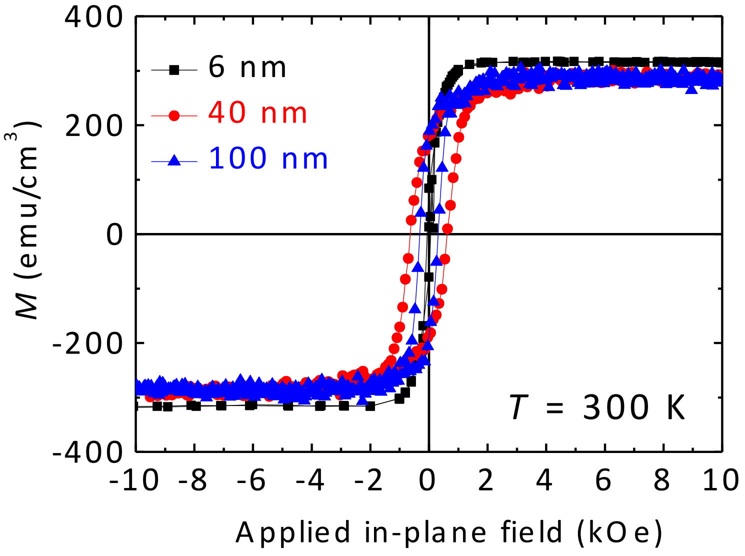

Supplementary Figure S2. Magnetic hysteresis loops at RT for thin films of Fe_3O_4 with the external magnetic field applied parallel to the sample surface. The 6-nm-thick Fe_3O_4 film corresponds to the growth conditions of sample B, while the 40- and 135-nm-thick films correspond to the growth conditions of sample C of the main manuscript.

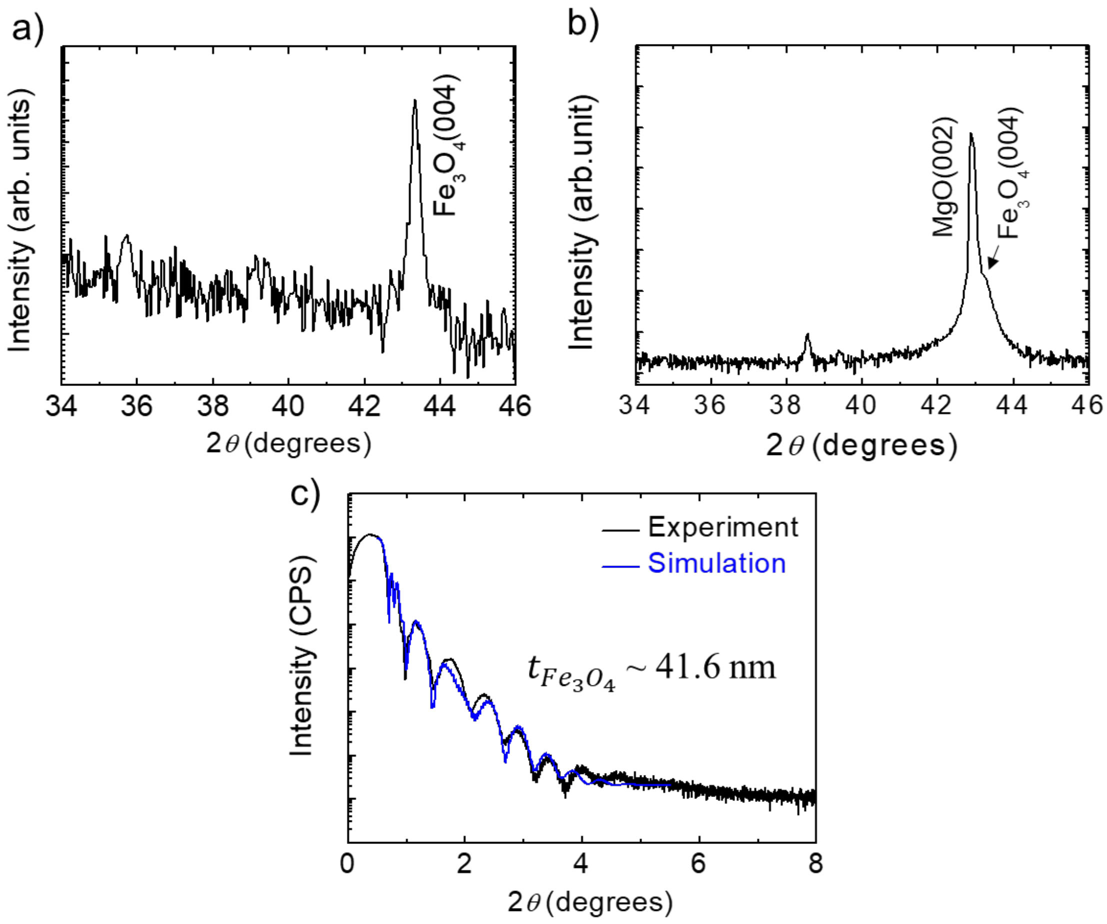

Supplementary Figure S3. X-ray diffraction of Fe_3O_4 films 100-nm-thick deposited by two different methods showing highly textured Fe_3O_4 (004) peaks for a) RF-magnetron sputtering and b) Oxide-MBE. c) X-ray reflectivity of a Fe_3O_4 film with nominal thickness of 40 nm deposited by RF-magnetron sputtering.

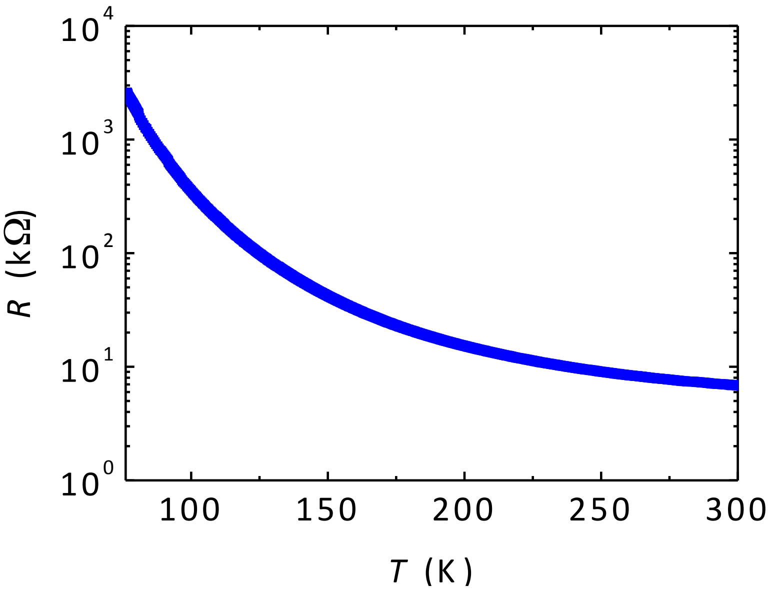

Supplementary Figure S4. Temperature dependence of the resistance of a 6-nm-thick Fe_3O_4 film as a function of temperature.

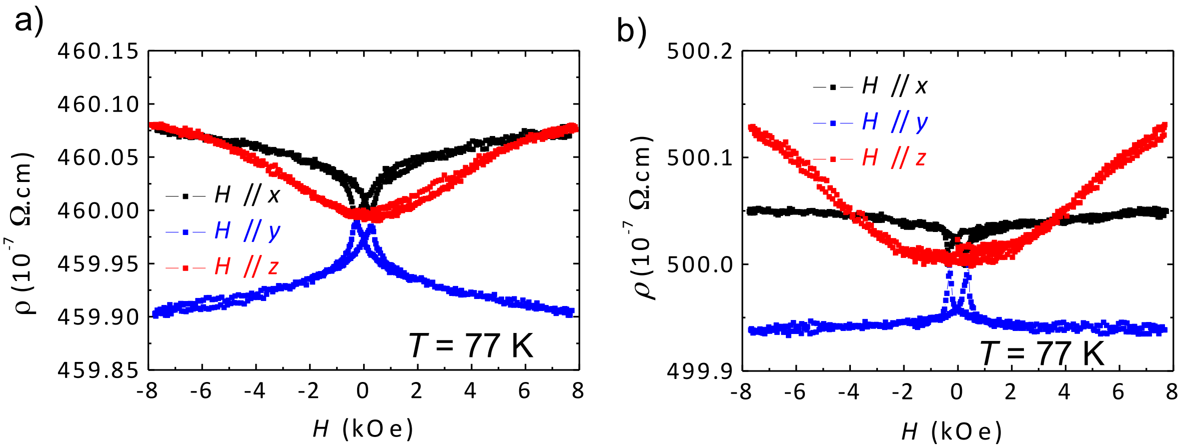

Supplementary Figure S5: (a) Magnetic field dependence of the resistivity of sample B, and (b) sample B1 at $T = 77$ K, for an external magnetic field applied along the *x*, *y* and *z*-axes.
